# Trend change analysis in the assessment of body balance during posture adjustment in reaction to anterior-posterior ground perturbation

**DOI:** 10.1371/journal.pone.0301227

**Published:** 2024-04-30

**Authors:** Piotr Wodarski, Marta Chmura, Michał Szlęzak, Grzegorz Bajor, Marek Gzik, Jacek Jurkojć

**Affiliations:** 1 Faculty of Biomedical Engineering, Department of Biomechatronics, Silesian University of Technology, Gliwice, Poland; 2 Fizjosport Medical Center, Gliwice, Poland; 3 Association of Neurophysiological-Orthopaedic Manipulative Physical Therapists, Gliwice, Poland; 4 Department of Human Anatomy, Medical University of Silesia, Slaskie, Poland; Opole University of Technology: Politechnika Opolska, POLAND

## Abstract

Postural adjustments (PA) occur to counteract predictable perturbations and can be impaired as a result of musculoskeletal and neurological dysfunctions. The most common way to detect PA is through electromyography measurements or center of pressure (COP) position measurements, where analysis in time domain and frequency domain are the most common. Aim of the research was to determine whether a new method of analyzing stabilographic measurements—the COP trend change analysis (TCI) of temporary posture corrections- can expand understanding of changes in balance strategy connected with PA. The study group involved 38 individuals (27women, 11men) aged 23±2.6 years. Measurements were performed using a stabilographic platform placed on a perturbation platform. The tests involved three measurements with forward and backward momentary movements of the platform. Participants were tested in three conditions–knowing the nature, time and direction of perturbation (Tr3), knowing only the nature of perturbation (Tr2) and without any information about the perturbation (Tr1). Statistically significant differences were revealed in the last second of Tr3 for the mean velocity of COP (p<0.05) and for two TCI parameters–TCI_dV (p<0.05) and TCI_dS (p<0.01). The increase in TCI_dV was related to the increase in the mean distance between trend changes (TCI_dS) and constant value of the mean time between trend changes (TCI_dT). The increase of the mean value of TCI_dS was the result of smaller number of posture corrections with the distance 0–2 mm and lager number with the distance 4–6 mm. Obtained results proved that the TCI analysis is a method enabling an extended analysis of PA, indicating the nature of changes occurring in posture corrections–longer momentary jumps of COP–related to a change in the strategy of maintaining balance before a known disorder, which has not been analyzed in this type of research so far.

## Introduction

Posture is not just about the simple, static positioning of body parts. It is a dynamic process that involves constant modifications to ensure balance while carrying out different activities. Postural adjustment (PA) and compensation are natural reactions of the body to destabilizing stimuli [1–3]. PAs can be early (EPAs) or anticipatory (APAs). Research conducted by Xie *et al*. [4,5], revealed that PAs may result from the internal initiation of movement (preparation to perform a certain activity). Similarly, Cleworth *et al*. [1] and Sibley and Etnier [6] indicated that a destabilizing stimulus may come from the environment. This conclusion was confirmed by Mohapatra and Krishnan [3], who also indicated correlations between the intensity of the APA and EPA and the intensity of the environmental stimulus effects. The tests performed by Ritzmann et al. [7] demonstrated the possibility of training PA resulting in faster post-perturbation balance recovery. Previous authors’ research [8] demonstrated the correlation between the intensity of the PA and the post-perturbation shift of the COP, which indicated that the PA mattered in the process of balance recovery.

PA can be observed by measuring changes in muscle activity, what is the most common method, but measurements of changes in the center of mass (COM) or the center of pressure (COP) displacements can also be used. In the case of COP measurements, PA is visible in the increase in COP velocity [9,10]. Other research revealed that the ranges of the COP and COM displacements during PA and compensation are directly correlated with the likelihood of falling [4,11,12].

Research conducted by Vuillerme and Nafati [13] revealed a change in the median of the main frequency of the COP movement during the concentration and preparation for an induced voice stimulus. The frequency of the COP movements can be analyzed when searching for cyclic changes using fast Fourier transform (FFT)-based algorithms [13–15], and wavelet analyses [16,17]. A new advancement in postural analysis is the application of trend change analysis (TCA). This method can identify rapid adjustments and an elongation in the displacement between consecutive postural adjustments [18,19]. Borrowed from strategies initially used in stock market analyses, TCA aids in measuring postural adjustments in both the front-to-back (A/P) and side-to-side (M/L) directions. Furthermore, it enables the computation of the count of adaptations and the time gap between successive posture adjustments, offering insights into how the body reacts to postural difficulties [20–22]. Such analysis may explain the origin of changes observed in other quantities, such as velocity as well as take into account non periodic postural correlations, which are not included in frequency analysis.

Taking the above into account, we hypothesize that the observed increase in velocity of COP is connected with the change of balance strategy, what should be visible in the changes in trend changes parameters in the COP signal, such as number of posture corrections as well as time and distance between following posture corrections. The aim of the study was to check if different conditions of PA examination would result in differences in values of trend changes parameters. Positive verification of the hypothesis may allow the formulation of a new method supporting assessment of PA. The reliability and validity of trend changes parameters was confirmed in previous research [18,19].

The tests presented in this article are the continuation of the research aimed at detecting the PA and accompanying phenomena related to COP movements, when preparing for postural perturbations connected with the abrupt movement of the ground [8]. These measurements made it possible to assess muscle activation in EPA and APA time intervals before movement of the basis.

## Materials and methods

### Study group

The study group consisted of 38 persons (27 women, 11 men) aged 23 ± 2.6 years, height 172 ± 9.6 cm and weight of 70 ± 17 kg. Excluding factors: past injuries of lower limbs and balance problems—both confirmed by physiotherapists and declared by potential participants. Each of the study participants gave written informed consent to participate in the study. This study had been previously approved by the Ethics in Research Committee of the Academy of Physical Education in Katowice (protocol number 5/2020).

### Experimental procedure

**Fig 1:**
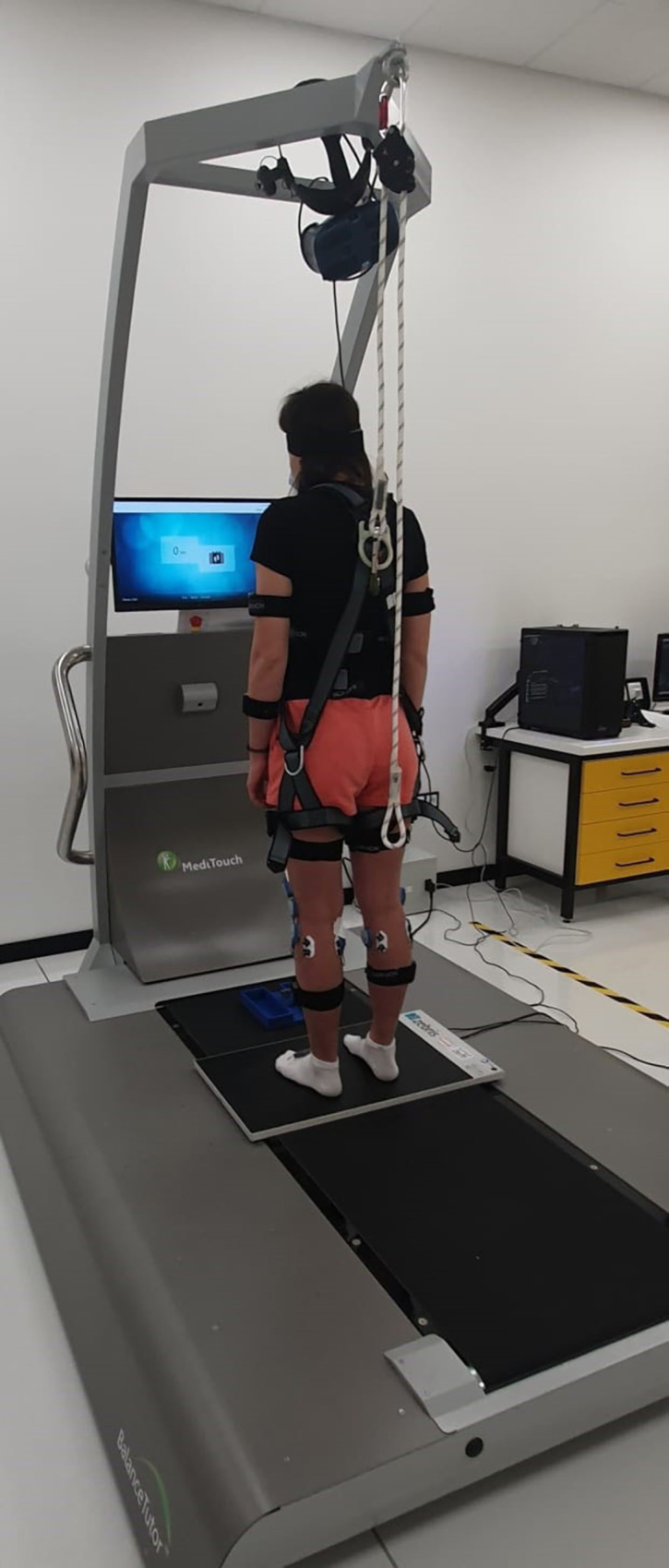
Measurement stand and a test participant.

The measurement stand was composed of a platform for the COP distribution measurements (WinFDM-S, Zebris, 100 Hz data acquisition frequency, 2560 tensiometer sensors, 34 cm x 54 cm sensors area) fixed to the central part of the perturbation platform in the form of the treadmill for the training and prevention of postural perturbations (BalanceTutor, MediTouch) (Fig 1). Pre-test preparations involved the attachment of the EMG electrodes (Ultium EMG, Noraxon, 2000 Hz data acquisition frequency) and inertial measurement unit (IMU) sensors (Ultium Motion, Noraxon, 200 Hz data acquisition frequency) [8]. Electrodes were attached to musculus tibialis anterior, musculus rectus femoris, musculus gastrocnemius medialis and musculus gastrocnemius lateralis—muscles actively involved in the process of maintaining balance in AP direction (a detailed description of the procedure for analyzing the recorded EMG signals along with the results can be found in the supplementary materials: S5 File). One IMU sensor was attached to the treadmill belt enabling the detection of platform motion initiation and synchronization of all devices.

A participant stood barefoot on a stabilographic platform protected against falling. Each study participant was protected against falling using a special harness attached above the balance platform (Fig 1). This device neither restricted movements nor affected their freedom. The participant’s task was quiet standing with arms along the sides during each of the measurements (Tr1,Tr2 and Tr3).

During individual measurements:

- Tr1 –participant did not know the nature, starting time, or direction of perturbations,
- Tr2 –participant knew the nature of perturbation but did not know its starting time and direction—participant was informed that the perturbation would be of the same nature as in the Tr1 trial,
- Tr3 –participant was informed (using a display) about the starting time and the direction of perturbation. The countdown was auditory and was also displayed 5 s before perturbation. The countdown intervals amounted to 1 s.

The same participants were involved in the three measurements (Tr1,Tr2 and Tr3). Each measurement lasted 30 seconds and consisted of two movements of the perturbation platform: the first one–forward–that took place 10 s after the initiation of measurements and the second one–backward– 20 s after the initiation of measurements. Both movements were identical, with a length of 9,5 cm and a duration of 0.5 s. The test was repeated three times. In case of balance loss the measurement was repeated.

### Processing of results

After taking the measurements the results were processed as follows:

- determination of perturbation platform movement initiation using the sensor attached to the treadmill belt—detection of the beginning of movement based on the detection of acceleration increase,
- determination of the COP displacements in the AP and ML directions—COP waveform exported from the measurement platform,
- signal filtration using a low-pass Butterworth IIR filter (Fpass = 10 Hz, Fstop = 12 Hz, Astop = -60 dB),
- division into sections lasting 1 s each, counting from the 6^th^ second preceding perturbation to the moment of platform perturbation.

For each 1-second section, the average velocity of the COP displacement was calculated (Vcop), and an analysis of trend changes was performed.

### Trend changes index calculation

The method of trend change analysis comes from technical analysis of signals, which is based on moving averages with exponential weights. To begin with, the algorithm employs the MACD (Moving Average Convergence Divergence) indicator calculation process in its initial phase [18,19]. This algorithm assesses relationships linked to the convergence and divergence of moving averages of a measured signal—in the study it is COP signal. In the initial calculation, it was determined the MACD line for the COP signal, by computing the difference between two Exponential Moving Averages (EMAs) with lengths of 12 and 26 samples, as per Eqs 1 and 2.

$$MACD={EMA}_{COP,12}-{EMA}_{COP,26}.$$ Eq 1

where:

EMACOP,12—faster exponential moving average for COP signal,

EMACOP,26—slower exponential moving average for COP signal.

$$EMA=\frac{p_{0}+{(1-\alpha )p}_{1}+{\left(1-\alpha \right)}^{2}p_{2}+\cdots +{\left(1-\alpha \right)}^{N}p_{N}}{1+(1-\alpha )+{\left(1-\alpha \right)}^{2}+\cdots +{\left(1-\alpha \right)}^{N}}.$$ Eq 2

where:

p0 –ultimate value,

p_1_ –penultimate value,

p_N_−value preceding N periods,

N = number of periods,

α = a smoothing coefficient equal to 2/(N+ 1).

Moving on to the subsequent phase, the signal line is calculated as an EMA with a length of 9 samples from the MACD line signal in accordance with Eq 3.


$$Signal\;line={EMA}_{MACD\;line,9}.$$ Eq 3

**Fig 2:**
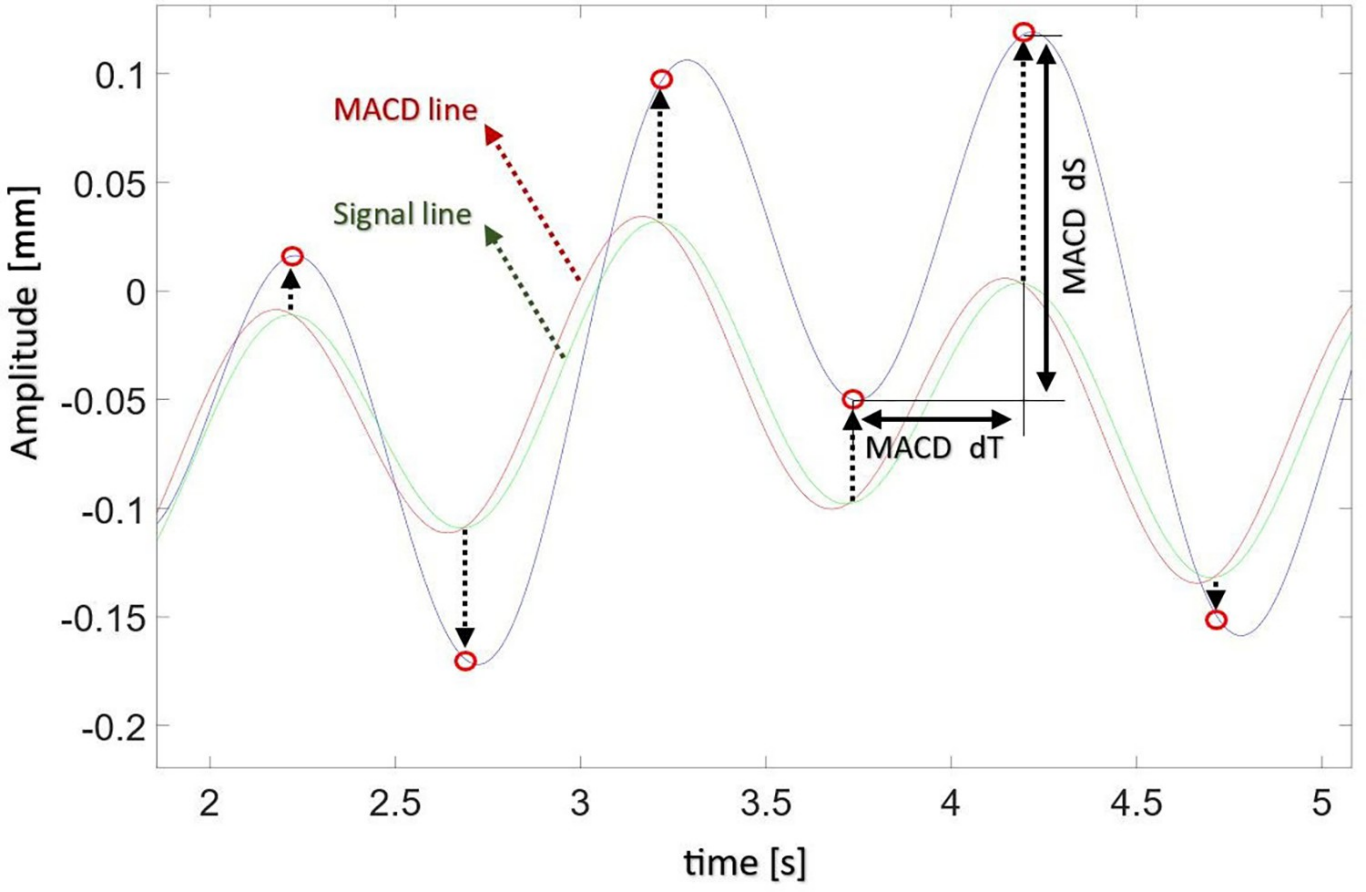
Graphical interpretation of determined parameters and places of trend changes calculated on basis of MACD algorithm. The blue line shows the signal course, the red circles mark the places of trend changes detected by the algorithm. The Signal line is marked in green and the MACD line is marked in red.

The points where the MACD line intersects with the Signal line are pivotal in identifying shifts in the trend of the COP signal (Fig 2). These intersections, in terms of quantity, determine what it refers to as the Trend Changes Index (TCI).

### Basic trend changes analyses

Moving forward, it was proceeded to calculate the time sections between successive trend change points in the COP signal (Fig 2). This process led to the creation of the MACD_dT table (table contains of detected MACD_dT values), whose average value for the entire analyzed course is denoted as TCI_dT. Consequently, we calculated the displacements between consecutive trend change points, resulting in the MACD_dS table. The average value of this table corresponds to TCI_dS. Lastly, the MACD_dV table is established by dividing the values in the MACD_dS table by their corresponding MACD_dT table. The TCI_dV indicator is then determined as the average value of the MACD_dV table.

The following quantities were determined separately for AP and ML directions:

- TCI–total number of trend changes during the entire test,
- TCI_dT–mean value of time between successive trend changes,
- TCI_dS–mean value of displacement between successive trend changes,
- TCI_dV–mean velocity of the COP displacement between successive trend changes.

Resultant values were calculated as follows:

- for TCI, results obtained for both directions were summed together,
- for other quantities, resultant value determined on the basis of the AP and ML components as the square root of the sum of squares of the values calculated in each direction.

The forward and backward displacements of the platform are analyzed as one group because the movement of the COP before the displacement is analyzed. Changes of feature of the COP signals are important in these analyzes and they can be manifested in the number of trend changes of the COP signals—these changes are related to the number of posture corrections, regardless of the direction in which this correction occurs.

### Histogram analysis

The more precise indication how increase in TCI_dS in the last second before the disturbance in the Tr3 test was corelated with changes in MACD_dS values required division into smaller displacement intervals of this quantity. This division was presented on histograms containing the number of individual MACD_dS’s (expressed as a percentage) in each displacement interval divided into subsequent time sections before the disturbance. The interval discretization step in the TCI_dS displacement histogram charts amounted to 2 mm, whereas results were presented up to a maximum displacement of 30 mm. The tests did not reveal TCI_dS values exceeding 30 mm. For each subject and each test, the TCI_dS histogram chart was calculated before perturbation. The diagrams present the median values obtained for the group of test participants. The values of changes (*i*.*e*., number of displacements) per each displacement interval are expressed as a percentage. The sum of all of the displacements in relation to each displacement interval subjected to analysis was adopted as 100%.

### Statistical analysis

The Shapiro-Wilk test did not reveal the presence of normal distributions in relation to all parameters, therefore, median values were used in all analyses. The Friedman test, followed by the Pairwise Wilcoxon with a post-hoc Holm correction test, were used to compare the parameters between the Tr1, Tr2, and Tr3 tests. In addition, the size of the effect was calculated using the methodology proposed by Rea and Parker [23]. The difference between the medians was reported as statistically significant as long as the effect was high (0.8 and more) or at least medium (0.5–0.8). Small effects were not reported.

## Results

Analyzes of the EMG signals obtained for the described tests, which were presented in detail in the article [9], showed differences in the activation of the lower limb muscles between trials Tr1, Tr2 and trial Tr3. The information that was used from the analysis was mainly: the presence of PA (in the form of APA and EPA) was objectively confirmed in the Tr3 trial and the time at which PA occurred in the EMG signal was determined, which, as it turned out, did not exceed one second before the disturbance. It was shown that in the Tr3 trial, EPA and then APA appear during the one second preceding the disturbance (S2 and S4 Files). This made it possible to indicate the time intervals of both these reactions–EPA and APA. It was assumed, that different muscle activations bring about different characteristics of COP signals in the detected time intervals, what should be visible in different values of analyzed quantities. Taking into account this assumption, analyzes of the COP movement were carried out for 6 seconds preceding the introduced perturbation assuming that PAs may be detected in the last seconds.

Results from the -6^th^ to the -1^st^ second (Table 1, S1 File) are identical (the TCI median is always an integer for an odd number of measurements). The TCI medians do not differ significantly between successive time sections in the Tr1, Tr2, and Tr3 tests.

**Table 1:** TCI values in successive moments before perturbation in tests Tr1, Tr2, and Tr3. The results concern the measurements from the 6^th^ second preceding perturbation (-6 s represents the time between the 6^th^ and the 5^th^ second before perturbation) to the moment of perturbation (-1 s represents the time between the 1 second preceding perturbation and the perturbation itself).

*Test*	*Tr1*						*Tr2*						*Tr3*					
*Time*	*-6 s*	*-5 s*	*-4 s*	*-3 s*	*-2 s*	*-1 s*	*-6 s*	*-5 s*	*-4 s*	*-3 s*	*-2 s*	*-1 s*	*-6 s*	*-5 s*	*-4 s*	*-3 s*	*-2 s*	*-1 s*
*Median*	*7*	*8*	*8*	*8*	*8*	*8*	*8*	*8*	*8*	*8*	*8*	*8*	*7*	*7*	*8*	*8*	*8*	*7*
*Quartile 1 (25%)*	*5*	*6*	*6*	*6*	*6*	*6*	*5*.*8*	*6*	*6*	*6*	*6*	*6*	*5*	*6*	*6*	*6*	*6*	*6*
*Quartile 3 (75%)*	*9*	*10*	*10*	*10*	*10*	*10*	*10*	*10*	*10*	*10*	*10*	*10*	*9*	*9*	*10*	*9*	*10*	*9*
*Mean*	*7*.*53*	*8*.*13*	*8*.*04*	*8*.*06*	*8*.*60*	*8*.*32*	*8*.*03*	*8*.*26*	*8*.*16*	*8*.*33*	*8*.*31*	*8*.*57*	*7*.*36*	*7*.*50*	*8*.*08*	*7*.*92*	*8*.*05*	*7*.*77*
*SD*	*3*.*12*	*3*.*05*	*2*.*71*	*3*.*19*	*3*.*10*	*2*.*82*	*3*.*17*	*2*.*98*	*3*.*09*	*2*.*93*	*3*.*04*	*2*.*94*	*2*.*74*	*2*.*74*	*2*.*55*	*2*.*96*	*3*.*22*	*2*.*65*

**Fig 3:**
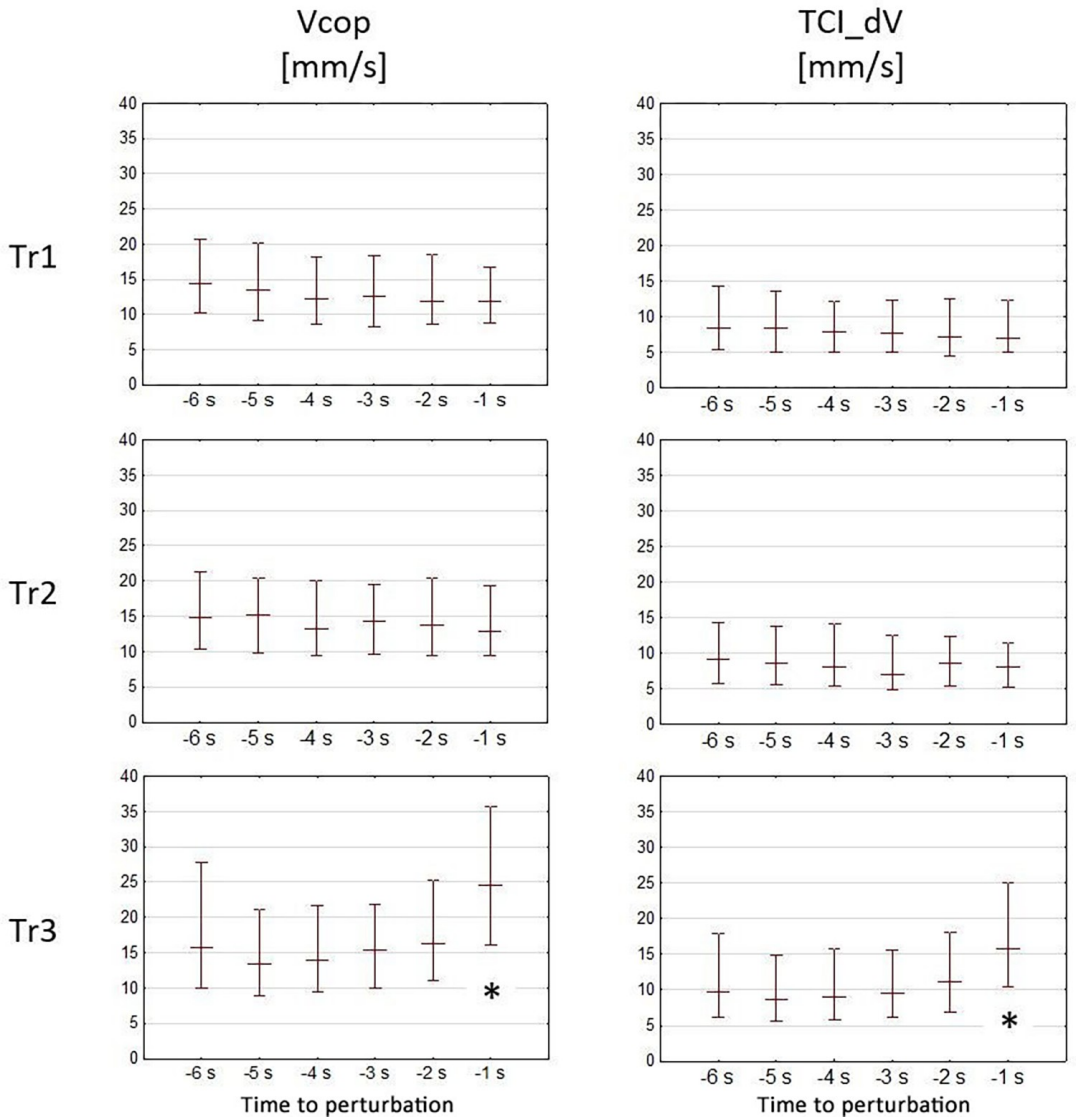
Values of Vcop and TCI_dV in relation to moments preceding perturbation in tests Tr1, Tr2, and Tr3. The chart presents the medians and the interquartile range for measurements between the 6^th^ second before perturbation (-6 s represents the time between the 6^th^ and the 5^th^ second preceding perturbation) to the moment of perturbation (-1 s represents the time between the 1 second before perturbation and the moment of perturbation). Statistically significant values differences (p < 0.05) are designated using “*”.

**Fig 4:**
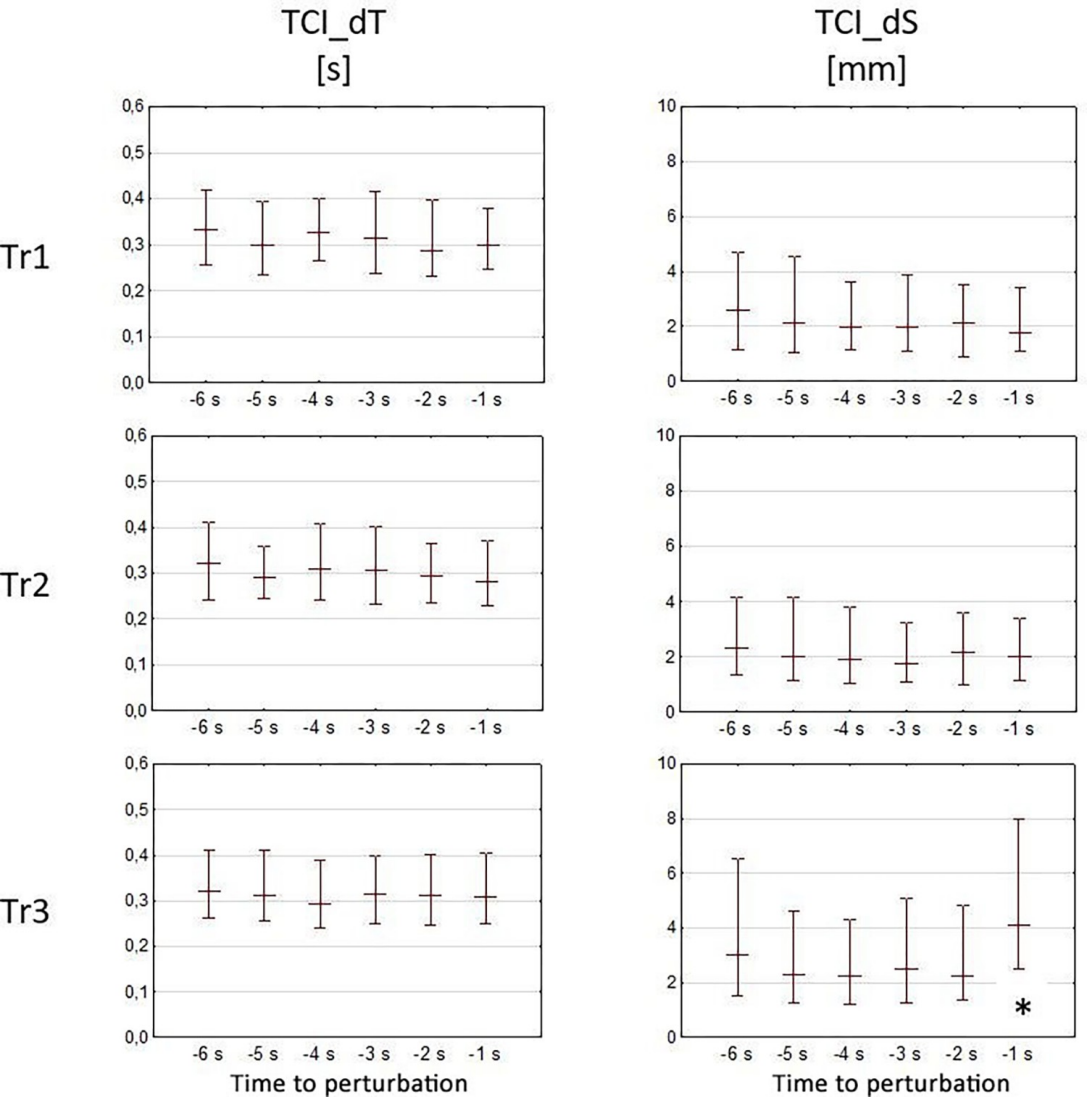
Values of TCI_dT and TCI_dS in relation to successive moments preceding perturbation in tests Tr1, Tr2, and Tr3. The chart presents the medians and the interquartile range for the measurements between the 6th second before perturbation (-6 s represents the time between the 6th and the 5th second preceding perturbation) to the moment of perturbation (-1 s represents the time between the 1 second before perturbation and the moment of perturbation). Statistically significant differences (p < 0.05) are designated using “*”.

In the case of TCI_dT, there were no statistically significant differences in the values between successive moments in all of the tests. However, in the case of TCI_dS (similar to TCI_dV and Vcop), it was possible to notice a significant increase in the distance (*p* < 0.05) in the Tr3 test at -1 s in relation to the measurements between -6 s and -2 s. A graphical presentations of the Vcop and TCI_dV values are presented in Fig 3, while the TCI_dt and TCI_ds values are presented in Fig 4.

**Fig 5:**
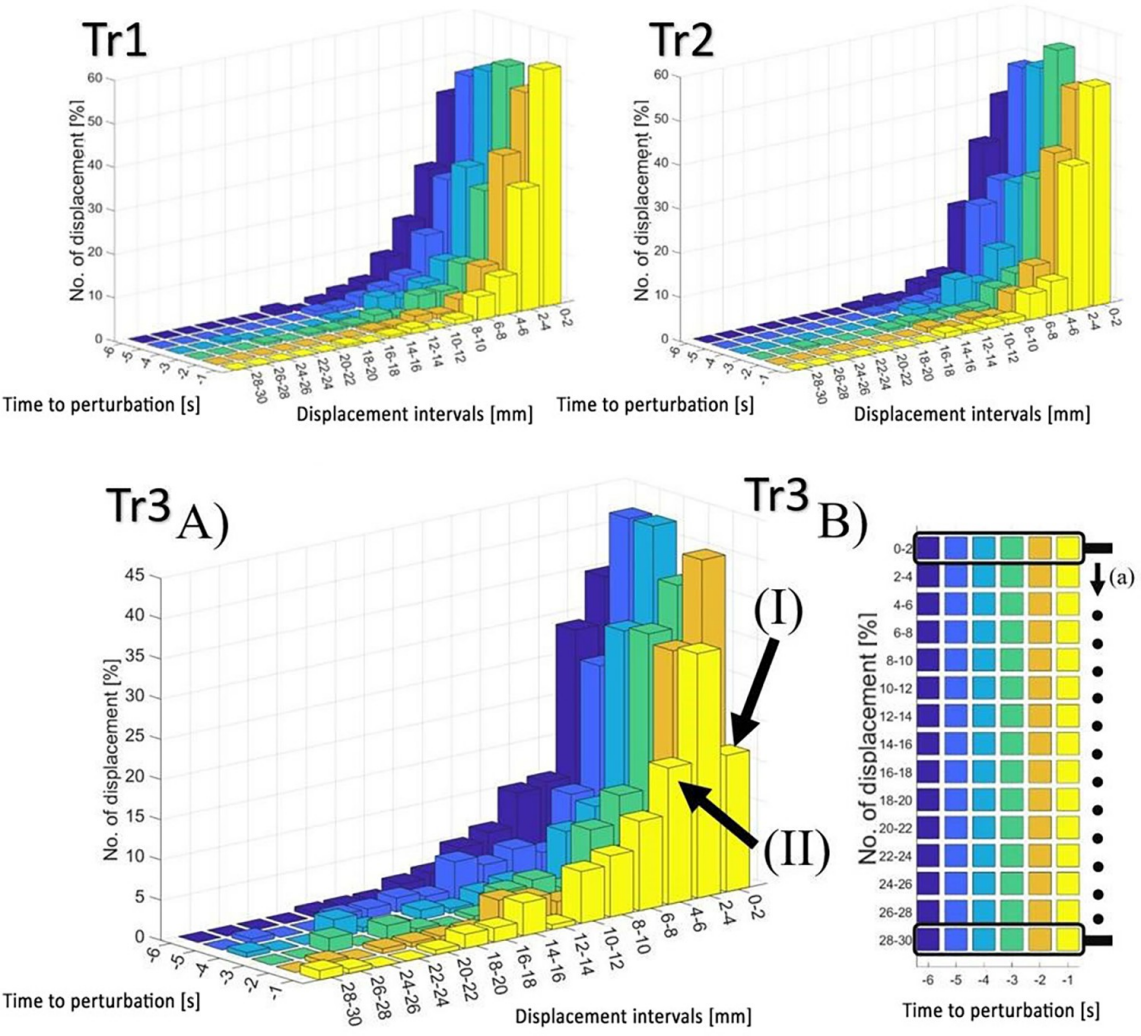
Histogram charts of TCI_dS in relation to Tr1, Tr2 and Tr3 trials. **The number of displacements is expressed as a percentage.** The sum of all of the displacements in relation to each displacement interval subjected to analysis was adopted as 100%. Tr3 A) Histogram charts of TCI_dS in relation to Tr3 at successive time sections preceding perturbation. The arrows I and II represent places of statistically significant changes in values between the time -1s and earlier time sections. Tr3 B) Top view of the TCI_dS histogram chart where the boxed-in areas designate the tests in relation to which the analysis involving the use of statistical methods was performed. The arrow (a) presents the sequence of analyses in related groups at increasing displacement intervals.

An increase in the TCI_dS values in the Tr3 test at -1 s significantly affected the change in TCI_dV. At the same time, an increase in the TCI_dS values also indicated a change in the balance-maintaining strategy at the aforesaid second of the movement. To further investigate the change of the strategy (in accordance with the methodology), histogram charts of TCI_dS at successive time sections preceding perturbation were plotted (Fig 5).

The number of displacements expressed as a percentage (median values, Fig 5) was subjected to the comparative analysis for each displacement interval. In all comparisons the ANOVA test was used to compare the medians of 15 groups of displacement interval in relation to all time sections and in accordance with the direction of the arrow “a” (Fig 5 Tr3 b) from the range of 0–2 mm to 28–30 mm (S3 and S6 Files). In each of the subgroups (where each subgroup contains values of number of displacements for all time intervals), it was possible to obtain several post-hoc comparisons. Statistically significant differences (α = 0.05) were obtained between 0–2 mm and 4–6 mm sections, indicated in Fig 5 Tr3 A) as arrow “I” and arrow “II”. The number of displacements in the displacement section 0–2 mm at -1 s time section (arrow I) in relation to the previous time periods (-6 s to -2 s) decreased (p<0.01). Conversely, the number of displacements in the displacement section 4–6 mm at -1 s (arrow II) in relation to previous time periods increased (p<0.01).

## Discussion

Muscle activity analysis is the basic method used to detect and analyze PA phenomena [8,22]. COP observation is used much more seldomly. Research conducted by Slijper *et al*. [24] and Krishnan *et al*. [25] are two examples that used COP analysis in PA detection. Both authors noticed an increase in the support path length in tests with suspected PA phenomenon. Bouisset [10] and Chander *et al*. [9] in their research indicated an increase in Vcop in relation to the baseline measurements. They also discovered that Vcop may be a better index to assess the PA phenomenon than COP displacements in AP and ML directions. All these observations were confirmed in described research.

Previous analyses [8] of the EMG signals obtained for these measurements proved the presence of EPA and APA in the Tr3 trial during the last second preceding the disturbance.

That was confirmed in current analyses of COP movement, where a statistically significant increase in Vcop was noticed in the last second preceding perturbation in Tr3 (Fig 3) which was not observed in tests Tr1 and Tr2. The similar increase can be observed in TCI_dV calculated from TCI_dT and TCI_dS what can confirm correctness of the assumptions of the developed method of trend changes analysis.

A surprising fact was the impossibility to observe changes in TCI numbers in subsequent time sections, particularly in test Tr3 (*i*.*e*., where the test participant knew when the moment of unbalance would take place). It would seem that increased Vcop and TCI_dV values should result in a different TCI number as a reaction of the nervous system aimed to coordinate the activity of individual muscles [26–29]. Additional analysis of TCI_dS and TCI_dT revealed that the observed increase in both velocities was primarily due to the extension of the path of momentary leaps of COP (TCI_dS) while maintaining constant times between leaps (TCI_dT) and consequently the same number of TCI.

What interesting opposite changes in balance strategy were observed in research comparing results obtained for people with Parkinson Disease and healthy participants during normal standing [18]. Measurements revealed higher values of Vcop and TCI_dV, just like in measurements described in this article, but smaller values of TCI_dT in PD group with no statistically significant differences in TCI_dS. This may indicate that there are at least two different mechanisms of adjusting the balance to destabilizing factors, possible to detect by means of trend change analysis–a physical disorder and, in mentioned research, Parkinson Disease.

Taking into account the fact that COP is a reaction to COM movement, registered, in this article, changes in TCI_dS could result from the change in the COM movement. However, taking into consideration the fact that the COP movements are identified as a resultant force acting the ground [30,31], it could also indicate the changed patterns of the work of muscles preparing for oncoming disruption, which is often observed in such types of tests [8,24,32,33]. However, the statement requires additional research.It should be noted that the lack of change in the number of trend changes could also result from the very short measurement time precluding the detection of slight differences.

### The balance-maintaining strategy based on the TCI_dS charts

Temporary posture corrections are one of the crucial elements related to the balance strategy. The developed TCI coefficient allows for a clear indication of the number of posture corrections. It can be expected that this number should be correlated with the average COP velocity, which, how it was mentioned above, was not observed in the conducted research. Despite the increase in Vcop in the last second before the perturbation in trial Tr3, the number of TCI in this interval remained unchanged. Observed differences in TCI_dS and lack of such changes in TCI_dT may affect the change in COP velocity, without the need to change the total number of trend changes (TCI). The research proved however that despite the constant value of total TCI, there are changes in number of COP displacements, but associated with a specific MACD_dS distance. It is presented on histograms for individual displacement intervals (Fig 5 –Tr3 A) where one can see a decrease of COP displacements in the firs displacement interval (0–2 mm interval at -1 second—Fig 5 Tr3, arrow I) and increase in the third interval (4–6 mm interval at -1 second—Fig 5 Tr3, arrow II). The observed differences indicate how participants tried to prepare for the upcoming move of the platform.

Taking into account previous analyses of authors and results available in other publications concerning the work of muscles [2,8,34], it can be assumed that the increase in muscle activity one second before the disturbance, resulting in an increase in the values of forces acting on the entire system, leads to an increase in the acceleration of COP movement and, consequently, an increase in the length of individual TCI_dS displacements without changes in TCI_dT. Possible correlation of the COP movement with the beginning of the platform movement—the direction of the COP movement consistent with the direction of the platform movement—could help maintain balance. The previous authors’ research [6] proved however that greater COP movements after disturbance were noted in trials when the subject knew the time and type of the introduced disturbance. This may indicate that the subject was more destabilized in those trials with known parameters of the disorder compared to trials when the time and type of the disorder were not known.

It can be assumed that awareness of the approaching movement of the platform and the resulting loss of balance leads to an increase in muscle tension, which only results in stiffening of the entire body. Such stiffening can be useful when one have to counteract the impact of an incoming object but does not help in the maintaining the balance on moving basis [35,36]. These results may support everyday observations that lack of awareness of an upcoming event results in fewer injuries in events such as falls or traffic accidents. Nevertheless, the above-presented conclusions require confirmation in further research.

## Conclusions

The article presents the possibilities of using the newly developed method of determining trend changes in COP movements in the study of PA phenomena as a complementary method to other COP analyses as well as EMG-based measurements. Determination of TCI parameters can show changes in the number of posture correcting movements. TCI_dS and TCI_dT as kinematic parameters can be used to explain other changes observed during such measurement, like increase in COP velocity, showing mechanism of the strategy of maintaining balance.

A significant novelty resulting from the use of the proposed method of analysis is information about the cause of changes in COP, which may result from a change in the number of posture corrections in time or from a change in the displacement between these posture corrections. Observation of changes in displacements in histograms not only allows for the detection of PA, it can also be a useful tool for measuring readiness in response to a stimulus, e.g. in sports training.

### Limitations

The proposed algorithm in the current version is not sensitive to very slow postural corrections. This is the result of a very short (on the order of single seconds in this study) time window and values of parameters in stock market algorithms used to calculate TCI values. The obtained results also require a thorough medical analysis, which could indicate links to the neurological aspect related to the strategies of maintaining balance.

## Supporting information

S1 File TCIres. Results of TCI calculation.
(XLSX)

S2 File APA. Results of APA calculation.
(XLSX)

S3 File dSTab. Analysis of TCI_dS histograms (MAT).
(XLS)

S4 File EPA. Results of EPA calculation.
(XLSX)

S5 File List of additional materials with description. (PDF)

S6 File Mmain 4D for dS. Matlab script to 4D charts visualizations (M).
(TXT)

## Abbreviations

Tr1: test no. 1 where the test participant did not know the nature, starting time, or direction of perturbation
Tr2: test no. 2, where the test participant knew the nature of perturbation but did not know its starting time and direction
Tr3: test no. 3, where the test participant was informed (using a countdown display) about the starting time and direction of perturbation
-6 s ….. -1 s: period of time preceding perturbation: - 6th second preceding perturbation (-6 s represents the time between the 6th and the 5th second before perturbation) to the moment of perturbation (-1 s represents the time between the 1 second preceding perturbation and the perturbation itself)
AP: anterior-posterior
ML: mediolateral
FFT: fast Fourier transform
COP: center of foot pressure
COM: center of mass
APA: anticipatory postural adjustment(s)
EPA: early postural adjustment(s)
Fpass: filter pass frequency
Fstop: filter cut-off frequency
TCI: total number of trend changes during the entire test
TCI_dT: mean value of time between successive trend changes
TCI_dS: mean value of displacement between successive trend changes
TCI_dV: mean value of the COP displacement velocity between successive trend changes
Vcop: resultant COP displacement velocity
IMU: inertial measurement unit
EMG: electromyography
