# Supplementary material for: Trend change analysis in the assessment of body balance during posture adjustment in reaction to anterior-posterior ground perturbation
Source: PLoS One. 2024 Apr 30;19(4):e0301227. doi: 10.1371/journal.pone.0301227 (PMC11060584; doi:10.1371/journal.pone.0301227)
Supplement: S5 File — (PDF) [file pone.0301227.s005.pdf]

List of additional materials with description

File list:

- **S2_APA_Results_of_APA_calculation.xlsx and S4_EPA_Results_of_EPA_ calculation.xlsx**

The files contain raw APA and EPA analysis results for the patients studied. The values included in the files are the results of the analysis described below. Additionally, mean values, medians, quartile range, standard deviation and coefficient of variation are included. The tables below the results contain the results of the p coefficient from the statistical analysis for each of the analyzed values, divided into groups and moments before the test.

The analysis was performed for the following muscles divided into right and left limb: musculus tibialis anterior (TA), musculus rectus femoris (RF), musculus gastrocnemius medialis (GM) and musculus gastrocnemius lateralis (GL)

Method for determining APA and EPA

At the first stage of the analyses based on the results obtained in the ERx test (during sitting on a chair), it was possible to identify the average resting activity of each of the muscles subjected to analysis (EMGRx). Afterwards, the activity of each muscle measured in Tr1, Tr2 and Tr3 were divided by the EMGRx value, performing the standardisation of measured parameters in relation to the values measured at rest.

Afterwards, on the basis of the data obtained from the IMU sensor located on the treadmill belt it was necessary to determine the beginning of the treadmill movement (t_0) for forward and backward movements.

The subsequent step aimed to answer the question whether information about the starting time and the direction of perturbation leads to an increase in the muscular tension of lower limb muscles. That stage required the investigation of the muscular activity of lower limbs in relation to APAs and EPAs in tests Tr1, Tr2 and Tr3. Time values and activity areas subjected to the analysis are presented in Fig.1.

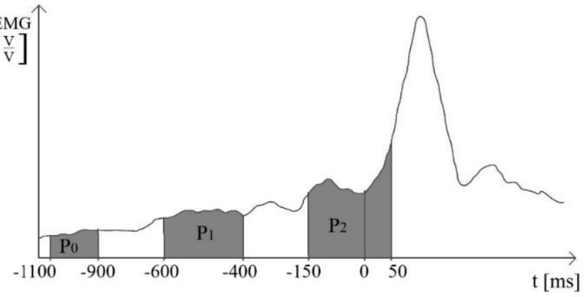

Fig. 1. Analysed time intervals of muscular activity. The vertical axis represents the multiplicity of the resting value assuming a symbolic value of V/V. The horizontal axis represents time in milliseconds (ms).

Area P0 stands for the area of muscular activity during free standing (between 1100 ms and 900 ms before perturbation). Area P1 represents the area of search for an increase in the muscular activity triggered by EPA (between 600 ms and 400 ms before perturbation). Area P2 represents the area of an increase in the muscular activity triggered by APAs (between 150 ms before perturbation and 50 ms after perturbation). The muscular activity related to APA and EPA (EMG_{APA} and EMG_{EPA},) was determined using formulas 1.1 – 1.5. The values of EMG_{APA} and EMG_{EPA} were identified for each of the muscles subjected to the tests.

1.1 $$P0 = \int_{-1100}^{-900} EMG\ dt$$

1.2 $$P1 = \int_{-600}^{-400} EMG\ dt$$

1.3 $$P2 = \int_{-150}^{50} EMG\ dt$$

1.4 $$EMG_{EPA} = P1 – P0$$

1.5 $$EMG_{APA} = P2 – P0$$

- **S1_TCIres_Results_of_TCI_calculation.xlsx**

The file contains two tabs in which the quantities included in the article were analyzed, such as: support path length, TCI, TCI_dT, TCI_dS and TCI_dV.

Mean values, medians, quartile range, standard deviation and coefficient of variation are included for each of the analyzed quantities divided into moments of time (7-1 s, the interval 7 s-6 s was additionally analyzed) and test conditions (Tr1, Tr2 and Tr3). The results are presented in tables. Additionally, p values resulting from statistical analysis for comparisons between moments of time are included.

- **S3_dSTab.xls and S6_Mmain_4D_for_dS.txt**

The mat file contains the results included in the analysis of TCI_dS histograms. Due to the three-dimensional structure of the data, the script Mmain_4D_for_dS.m was created for the results, which shows how to interpret the data and allows you to generate the charts included in the article.
